# Genetic variants of glutamate receptor gene family in Taiwanese Kawasaki disease children with coronary artery aneurysms

**DOI:** 10.1186/2045-3701-4-67

**Published:** 2014-11-19

**Authors:** Ying-Ju Lin, Jeng-Sheng Chang, Xiang Liu, Hsinyi Tsang, Ting-Hsu Lin, Chiu-Chu Liao, Shao-Mei Huang, Wen-Kuei Chien, Jin-Hua Chen, Jer-Yuarn Wu, Chien-Hsiun Chen, Li-Ching Chang, Cheng-Wen Lin, Tsung-Jung Ho, Fuu-Jen Tsai

**Affiliations:** Department of Medical Research, China Medical University Hospital, Taichung, Taiwan; School of Chinese Medicine, China Medical University, Taichung, Taiwan; Department of Pediatrics, China Medical University Hospital, Taichung, Taiwan; National Institute of Allergy and Infectious Diseases, National Institutes of Health, Bethesda, Maryland USA; Biostatistics Center, China Medical University, Taichung, Taiwan; Biostatistics Center and School of Public Health, Taipei Medical University, Taipei, Taiwan; Institute of Biomedical Sciences, Academia Sinica, Taipei, Taiwan; Department of Medical Laboratory Science and Biotechnology, China Medical University, Taichung, Taiwan; Division of Chinese Medicine, China Medical University Beigang Hospital, Yunlin County, Taiwan; Division of Chinese Medicine, Tainan Municipal An-Nan Hospital -China Medical University, Tainan, Taiwan; Asia University, Taichung, Taiwan

**Keywords:** KD, *GRIK1*, Single nucleotide polymorphism, CAA

## Abstract

**Background:**

Patients with Kawasaki disease (KD), a pediatric systemic vasculitis, may develop coronary artery aneurysm (CAA) as a complication. To investigate the role of glutamate receptors in KD and its CAA development, we performed genetic association studies.

**Methods and results:**

We examined the whole family of glutamate receptors by genetic association studies in a Taiwanese cohort of 262 KD patients. We identified glutamate receptor ionotropic, kainate 1 (*GRIK1*) as a novel susceptibility locus associated with CAA formation in KD. Statistically significant differences were noted for factors like fever duration, 1st Intravenous immunoglobulin (IVIG) used time (number of days after the first day of fever) and the *GRIK1* (rs466013, rs425507, and rs38700) genetic variants. This significant association persisted even after using multivariate regression analysis (Full model: for rs466013: odds ratio =2.12; 95% CI =1.22-3.65; for rs425507: odds ratio =2.16; 95% CI =1.26-3.76; for rs388700: odds ratio =2.16; 95% CI =1.26-3.76).

**Conclusions:**

We demonstrated that *GRIK1* polymorphisms are associated CAA formation in KD, even when adjusted for fever duration and IVIG used time, and may also serve as a genetic marker for the CAA formation in KD.

**Electronic supplementary material:**

The online version of this article (doi:10.1186/2045-3701-4-67) contains supplementary material, which is available to authorized users.

## Background

Patients with Kawasaki disease (KD), an acute systemic vasculitis, may develop coronary artery aneurysm (CAA) as a complication. KD is one of the leading causes of acquired cardiovascular diseases in childhood. Infectious agents, host immune dysregulation, and genetic susceptibility are thought to be responsible for the development of KD and its related complications[1–3]. However, the pathological mechanisms underlying KD remain to be elucidated.

Numerous genome-wide association studies have been conducted to identify host cellular genes that affect KD susceptibility[4–14] in the European, Japanese, Korean, and Taiwanese populations. In the European population[11, 13], no common SNPs have been identified as susceptibility loci for European KD. However, a common SNP (rs2233152; *MIA* gene) was observed in the European, Japanese, and Taiwanese populations[9–11]. Common gene SNPs among Asians including Japanese, Taiwanese, and Korean populations have also been observed[4, 6, 9, 10, 12, 14, 15]. Six SNPs, namely, rs2736340 (*BLK*), rs2618479 (*BLK*), rs6993775 (*BLK*), rs10401344 (*ITPKC*), rs2233152 (*MIA*), and rs4813003 (*CD40*) have been observed in both Japanese and Taiwanese populations[9, 10] (Additional files1 and2). These studies suggest that genes involved in the immune-regulatory responses and cardiovascular-related pathogenesis may contribute to KD susceptibility.

Glutamate receptors were initially demonstrated to play important roles in excitatory neurotransmission in the brain and interneuronal communication[16]. Based on their different activation mechanisms, glutamate receptors can be divided into 2 groups: ionotropic glutamate receptors (iGluRs) and metabotropic glutamate receptors (mGluRs). The human genome is known to contain at least 16 iGluRs and 8 mGluRs. Based on their agonist binding and electrophysiological properties, iGluRs can be classified to 3 groups: alpha-amino-3-hydroxy-5-methyl-4-isoxazole (AMPA), N-methyl-D-aspartate (NMDA), and kainate (KA) receptors. Genetic mutations in glutamate receptors are associated with a number of human diseases including autism, Huntington’s disease and Parkinson’s disease[17, 18]. In addition, glutamate receptors have been found to influence autoantigen/antibody interactions and multiple sclerosis. GluR3 (GRIA3) is known to act as an autoantigen in Rasmussen’s encephalitis, suggesting a strong link between glutamate receptors and autoimmune interaction in certain degenerative diseases[19]. The regulation of glutamate receptor binding activity can reduce central nervous system (CNS) inflammation, apoptosis, and axonal damage[20]. In addition, glutamate receptors have also been implicated in cardiovascular diseases[21]. Glutamate receptor 1 (GluR1), an AMPA receptor subtype, can mediate the regulation of platelet activation through glutamate and GluR1 knockout mice develop *in vivo* thrombosis after a prolonged time[22]. The activation of GluR1 may contribute to the development of cardiovascular disease via accelerating thrombus formation.

Endothelial cells are principal targets for ischemic free-radical injury. Glutamate receptors are known to prevent nitric oxide-induced vascular injury[23]. On the other hand, activation of certain glutamate receptors was demonstrated to be a potential strategy for disrupting angiogenesis[24]. Coronary artery damage in KD is strongly associated with endothelial cell dysfunction[25]. Additional evidence suggests that glutamate receptors may influence KD pathogenesis[26, 27].

To explore the role of glutamate receptors in KD development, we investigated the entire family of glutamate receptors by performing genetic association studies on a Taiwanese cohort of 262 KD patients. Our study identified *glutamate receptor ionotropic*, *kainate 1* (*GRIK1*) as a novel susceptibility locus on 21q21.3. To our knowledge, this is the first instance to screen the glutamate receptor family for the association between genetic variants of glutamate receptors and CAA formation in KD.

## Results

### Genetic association study of the glutamate receptor gene family in Taiwanese KD children and controls

To identify KD susceptibility genes, a total of 53 SNPs of 16 genes within the glutamate receptor gene family including *GRIK1*, *GRIK2*, *GRIK3*, *GRIK4*, *GRIK5*, *GRIA1*, *GRIA2*, *GRIA4*, *GRM1*, *GRM2*, *GRM3*, *GRM4*, *GRM5*, *GRM6*, *GRM7*, and *GRM8* genes were genotyped in 262 Taiwanese KD children and in 1107 healthy people from the general population of Taiwan who were Han Chinese ethnic background for the SNP association study (Table 1). No significant differences were found between these 2 groups, suggesting that the glutamate receptor family genes may not contribute to KD susceptibility.

**Table 1:** **Genotype distribution of glutamate receptor family gene SNPs in Taiwanese KD patients and controls**

SNP	Chromosome	Cytoband	Physical position	Nearest genes		Controls	KD patients		
						No. (%)	No. (%)	***p***value	Odds ratio (95% CI)
rs466013	21	q21.3	29826390	*GRIK1*	TT + TC	507 (45.9)	131 (50.2)	0.205	1.19 (0.91-1.56)
					CC	599 (54.1)	130 (49.8)		1
rs425507	21	q21.3	29827658	*GRIK1*	GG + GA	507(45.8)	130 (49.6)	0.265	1.17 (0.89-1.53)
					AA	600 (54.2)	132 (50.4)		1
rs388700	21	q21.3	29830158	*GRIK1*	TT + TA	506 (45.7)	130 (49.6)	0.254	1.17 (0.89-1.53)
					AA	601 (54.3)	132 (50.4)		1
rs402280	21	q21.3	29835401	*GRIK1*	TT + TA	424 (38.3)	116 (44.3)	0.075	1.28 (0.97-1.68)
					AA	683 (61.7)	146 (55.7)		1
rs17816480	6	q16.3	101522140	*GRIK2*	TT + TC	201 (18.2)	48 (18.3)	0.951	1.01 (0.71-1.43)
					CC	906 (81.8)	214 (81.7)		1
rs2786239	6	q16.3	101637565	*GRIK2*	GG + GA	186 (16.8)	45 (17.2)	0.885	1.03 (0.72-1.47)
					AA	921 (83.2)	217 (82.8)		
rs4840194	6	q16.3	101768497	*GRIK2*	CC + CT	357 (32.2)	88 (33.6)	0.677	1.06 (0.80-1.41)
					TT	750 (67.8)	174 (66.4)		1
rs1310715	6	q16.3	101961427	*GRIK2*	TT + TC	597 (53)	133 (50.9)	0.468	0.91 (0.69-1.91)
					CC	520 (47.0)	128 (49.1)		1
rs527631	1	p34.3	36844396	*GRIK3*	AA + AG	172 (15.5)	45 (17.6)	0.407	1.16 (0.81-1.67)
					GG	935 (84.5)	210 (82.4)		1
rs476894	1	p34.3	36868682	*GRIK3*	GG + GA	234 (21.1)	63 (24.0)	0.305	1.18 (0.86-1.62)
					AA	873 (78.9)	199 (76.0)		1
rs541671	1	p34.3	36905238	*GRIK3*	TT + TA	267 (24.1)	65 (25.9)	0.554	1.10 (0.80-1.51)
					AA	840 (75.9)	186 (74.1)		1
rs35317705	1	p34.3	36972969	*GRIK3*	CC + CT	128 (11.6)	33 (12.6)	0.641	1.10 (0.73-1.66)
					TT	979 (88.4)	229 (87.4)		1
rs11218005	11	q23.3	120782227	*GRIK4*	AA + AC	132 (11.9)	35 (13.4)	0.523	1.14 (0.76-1.70)
					CC	975 (88.1)	227 (86.6)		1
rs3901285	11	q23.3	120862726	*GRIK4*	TT + TC	650 (58.7)	158 (60.3)	0.638	1.07 (0.81-1.41)
					CC	457 (41.3)	104 (39.7)		1
rs4936566	11	q23.3	120944529	*GRIK4*	AA + AG	669 (60.4)	145 (55.3)	0.131	0.81 (0.62-1.06)
					GG	438 (39.6)	117 (44.7)		1
rs443239	19	q13.2	42001892	*GRIK5*	CC + CG	289 (26.1)	64 (24.4)	0.576	0.91 (0.67-1.25)
					GG	818 (73.9)	198 (75.6)		1
rs1493395	5	q33.2	153532297	*GRIA1*	AA + AG	565 (51.1)	125 (47.7)	0.326	0.87 (0.67-1.14)
					GG	541 (48.9)	137 (52.3)		1
rs12153489	5	q33.2	153568777	*GRIA1*	CC + CT	1087 (98.2)	259 (98.9)	0.454	1.59 (0.47-5.39)
					TT	20 (1.8)	3 (1.1)		1
rs4424038	5	q33.2	153740704	*GRIA1*	CC + CT	1102 (99.5)	262 (100.0)	0.276	ND
					TT	5 (0.5)	0 (0.0)		1
rs17035909	4	q32.1	157247565	*GRIA2*	AA + AT	351 (31.7)	87 (33.3)	0.640	1.07 (0.80-1.43)
					TT	756 (68.3)	175 (66.7)		1
rs17035959	4	q32.1	157302204	*GRIA2*	AA + AC	1075 (97.1)	255 (97.3)	0.848	1.08 (0.47-2.48)
					CC	32 (2.9)	7 (2.7)		1
rs7695870	4	q32.1	157342624	*GRIA2*	CC + CT	1082 (97.7)	258 (98.5)	0.460	1.49 (0.51-4.32)
					TT	25 (2.3)	4 (1.5)		1
rs6855973	4	q32.1	157365463	*GRIA2*	AA + AT	1085 (98)	258 (98.4)	0.623	1.31 (0.45-3.83)
					TT	22 (2.0)	4 (1.6)		1
rs10895875	11	q22.3	105785485	*GRIA4*	AA + AT	715 (64.6)	181 (69.1)	0.169	1.23 (0.92-1.64)
					TT	392 (35.4)	81 (30.9)		1
rs4754136	11	q22.3	105846312	*GRIA4*	CC + CT	1102 (99.5)	261 (99.6)	0.877	1.18 (0.14-10.18)
					TT	5 (0.5)	1 (0.4)		1
rs17104835	11	q22.3	105971356	*GRIA4*	CC + CT	447 (40.4)	104 (39.8)	0.839	0.97 (0.74-1.28)
					TT	660 (59.6)	158 (60.2)		1
rs7750018	6	q24.3	146206595	*GRM1*	CC + CT	285 (25.7)	62 (23.7)	0.486	0.89 (0.65-1.23)
					TT	822 (74.3)	200 (76.3)		1
rs362851	6	q24.3	146389448	*GRM1*	CC + CG	713 (64.4)	169 (64.5)	0.977	1.00 (0.76-1.33)
					GG	394 (35.6)	93 (35.5)		1
rs2300631	6	q24.3	146428918	*GRM1*	AA + AG	828 (74.8)	192 (63.3)	0.613	0.92 (0.68-1.25)
					GG	279 (25.2)	70 (26.7)		1
rs12023603	3	p21.2	51466999	*GRM2*	AA + AG	1076 (97.2)	253 (96.6)	0.583	0.81 (0.38-1.72)
					GG	31 (2.8)	9 (3.4)		1
rs1983842	3	p21.2	51535259	*GRM2*	AA + AG	1070 (96.7)	253 (96.6)	0.940	0.97 (0.46-2.04)
					GG	37 (3.3)	9 (3.4)		1
rs802441	7	q21.11	86657787	*GRM3*	CC + CT	1081 (97.6)	255 (97.3)	0.759	0.88 (0.38-2.04)
					TT	26 (2.4)	7 (2.7)		1
rs802466	7	q21.11	86698122	*GRM3*	CC + CT	222 (20.0)	44 (16.8)	0.230	0.80 (0.56-1.15)
					TT	885 (80.0)	218 (83.2)		
rs12704286	7	q21.11	86745625	*GRM3*	AA + AG	364 (32.9)	91 (34.8)	0.567	1.09 (0.82-1.44)
					GG	743 (67.1)	171 (65.2)		1
rs17697415	7	q21.11	86772500	*GRM3*	AA + AG	138 (12.5)	34 (13.0)	0.822	1.05 (0.70-1.57)
					GG	969 (87.5)	228 (87.0)		1
rs1873254	6	p21.31	34058712	*GRM4*	AA + AG	518 (55.9)	150 (57.4)	0.096	1.26 (0.96-1.66)
					GG	488 (44.1)	112 (42.6)		1
rs937039	6	p21.31	34075875	*GRM4*	AA + AG	1090 (98.5)	260 (99.2)	0.337	2.03 (0.47-8.83)
					GG	17 (1.5)	2 (0.8)		1
rs1565361	6	p21.31	34089248	*GRM4*	CC + CT	503 (45.5)	117 (44.7)	0.819	0.97 (0.74-1.27)
					TT	604 (54.5)	145 (55.3)		1
rs4106126	11	q14.2	88647181	*GRM5*	CC + CT	1093 (98.7)	256 (97.7)	0.214	0.55 (0.21-1.44)
					TT	14 (1.3)	6 (2.3)		1
rs1391878	11	q14.2	88713212	*GRM5*	CC + CT	264 (23.9)	58 (22.1)	0.557	0.91 (0.66-1.25)
					TT	843 (76.1)	204 (77.9)		1
rs12787863	11	q14.2	88810547	*GRM5*	AA + AG	447 (40.4)	110 (42.0)	0.634	1.07 (0.81-1.40)
					GG	660 (59.6)	152 (58.0)		1
rs7126679	11	q14.2	89020677	*GRM5*	AA + AG	651 (58.9)	160 (61.1)	0.513	1.10 (0.83-1.44)
					GG	455 (41.1)	102 (38.9)		1
rs2856354	5	q35.3	178978728	*GRM6*	AA + AG	1055 (95.3)	244 (93.1)	0.151	0.67 (0.38-1.16)
					GG	52 (4.7)	18 (6.9)		1
rs10464073	5	q35.3	178982284	*GRM6*	AA + AG	1056 (95.4)	244 (93.1)	0.132	0.65 (0.38-1.14)
					GG	51 (4.6)	18 (6.9)		1
rs17078880	5	q35.3	178983436	*GRM6*	CC + CT	1089 (98.4)	258 (98.5)	0.908	1.07 (0.36-3.18)
					TT	18 (1.6)	4 (1.5)		1
rs2645341	5	q35.3	178984314	*GRM6*	AA + AG	1087 (98.2)	258 (98.5)	0.756	1.19 (0.40-3.50)
					GG	20 (1.8)	4 (1.5)		1
rs6764411	3	p26.1	7101864	*GRM7*	AA + AC	927 (83.8)	211 (80.5)	0.202	0.80 (0.57-1.13)
					CC	179 (16.2)	51 (19.5)		1
rs17697928	3	p26.1	7326084	*GRM7*	AA + AG	861 (77.7)	199 (80)	0.525	0.90 (0.66-1.24)
					GG	246 (22.3)	51 (19.5)		1
rs779741	3	p26.1	7541915	*GRM7*	AA + AC	917 (82.8)	218 (83.2)	0.886	1.03 (0.72-1.47)
					CC	190 (17.2)	44 (16.8)		1
rs1354405	3	p26.1	7690304	*GRM7*	AA + AG	1012 (91.4)	236 (90.1)	0.491	0.85 (0.54-1.34)
					GG	95 (8.6)	26 (9.9)		1
rs712723	7	q31.33	126439090	*GRM8*	CC + CT	698 (63.0)	174 (66.4)	0.309	1.16 (0.87-1.54)
					TT	409 (37.0)	88 (33.6)		1
rs17627206	7	q31.33	126793483	*GRM8*	AA + AG	110 (9.9)	28 (10.7)	0.717	1.08 (0.70-1.68)
					GG	997 (90.1)	234 (89.3)		1
rs11563505	7	q31.33	127059729	*GRM8*	CC + CT	1086 (98.1)	258 (98.5)	0.687	1.25 (0.42-3.66)
					TT	21 (1.9)	4 (1.5)		1

GRIK, glutamate receptor, ionotropic, kainate; GRIA, glutamate receptor, ionotropic, AMPA; GRM, glutamate receptor, metabotropic, SNP, single nucleotide polymorphism; CI, confidence interval.

*p*-values were obtained by chi-square test (2 x 2 table).

Statistical significance was considered as *p* value <0.05.

Physical position of individual SNPs was based on the NCBI Assembly database: GRCH38 version.

### *GRIK1*genetic polymorphisms may be related to KD-associated CAA complications

**Figure 1:**
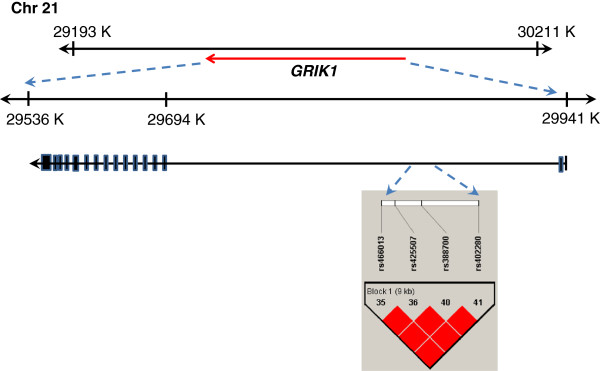
**Analysis of single nucleotide polymorphisms (SNPs) and the linkage disequilibrium (LD) pattern of the** ***GRIK1*** **gene.** Genomic location of SNPs present on chromosome 21q21.3. Physical position of individual SNPs was based on the NCBI Assembly database: GRCh38 version. Linkage disequilibrium (LD) blocks in the *GRIK1* gene, estimated by using HAPLOVIEW software. Pairwise D’ values (%) are indicated in squares; red indicates linkage disequilibrium [D’ =1, logarithm of odds (LOD) ≥2].

To examine the role of glutamate receptors in KD-associated CAA complications, we analyzed the correlation between KD children and the whole glutamate gene family. As shown in Table 2, the genotype distributions (dominant model) of 6 glutamate gene SNPs were statistically different between these 2 groups (*p* <0.05). These SNPs were rs466013, rs425507, rs388700, rs402280, rs17104835 and rs712723. Among these, 4 SNPs were found to be located in the *GRIK1* gene (*p* =0.007, 0.005, 0.004 and 0.022, respectively) (Additional file1). *GRIK1* consists of 18 exons and is located at 21q21.3 as shown in Figure 1. All SNPs were in Hardy-Weinberg equilibrium and had a successful genotyping frequency of >99%. The linkage disequilibrium (LD) structure of this region was also established, with 1 haplotype block determined. Four SNPs were located in that block. To evaluate the relationship among these 4 SNPs, pairwise LD analysis was performed. The D’ statistics were all 1.0. Strong LD was observed in the following 2 groups of SNPs, group1 (rs466013, rs425507, rs388700), with the *r*^2^ statistics >0.5 between every 2 SNPs in each group (data not shown). The frequencies of the TT and TC genotypes of *GRIK1* (rs466013) were significantly higher in KD patients with CAA than those in patients without CAA (63.2% for KD with CAA and 44.9% for KD without CAA complications; odds ratio =2.11 [95% confidence interval (CI) =1.22-3.65]). Similar results were also observed in rs425507, rs388700 and rs402280. These data suggest that *GRIK1* may be a potential susceptibility locus involved in the development of KD with CAA complications.

**Table 2:** **Association of the genetic variants of glutamate receptor family genes in Taiwanese KD children according to the presence or absence of CAA**

SNP	Chromosome	Cytoband	Physical position	Nearest genes	KD CAA-		KD CAA+		
						No. (%)	No. (%)	***p***value	Odds ratio (95% CI)
rs466013	21	q21.3	29826390	*GRIK1*	TT + TC	83 (44.9)	48 (63.2)	***0.007***	***2.11*** **(** ***1.22***-***3.65*** **)**
					CC	102 (55.1)	28 (36.8)		***1***
rs425507	21	q21.3	29827658	*GRIK1*	G + GA	82 (44.1)	48 (63.2)	***0.01***	***2.17*** **(** ***1.26***-***3.76*** **)**
					AA	104 (55.9)	28 (36.8)		***1***
rs388700	21	q21.3	29830158	*GRIK1*	TT + TA	81 (44.1)	48 (63.2)	***0.004***	***2.20*** **(** ***1.27***-***3.81*** **)**
					AA	104 (55.9)	28 (36.8)		***1***
rs402280	21	q21.3	29835401	*GRIK1*	TT + TA	74 (39.8)	42 (55.2)	***0.022***	***1.87*** **(** ***1.09***-***3.21*** **)**
					AA	112 (60.2)	34 (44.8)		***1***
rs17816480	6	q16.3	101522140	*GRIK2*	TT + TC	30 (16.1)	18 (23.7)	0.151	1.61 (0.84-3.11)
					CC	156 (83.9)	58 (76.3)		1
rs2786239	6	q16.3	101637565	*GRIK2*	GG + GA	29 (15.6)	16 (21.1)	0.288	1.44 (0.73-2.85)
					AA	157 (84.4)	60 (78.9)		1
rs4840194	6	q16.3	101768497	*GRIK2*	CC + CT	64 (34.4))	24 (31.6)	0.660	0.88 (0.50-1.56)
					TT	122 (65.6)	52 (68.4)		1
rs1310715	6	q16.3	101961427	*GRIK2*	TT + TC	91 (49.2)	42 (55.3)	0.373	1.28 (0.75-2.18)
					CC	94 (50.8)	34 (44.7)		1
rs527631	1	p34.3	36844396	*GRIK3*	AA + AG	32 (17.6)	13 (17.8)	0.966	1.02 (0.50-2.07)
					GG	150 (82.4)	60 (82.2)		1
rs476894	1	p34.3	36868682	*GRIK3*	GG + GA	45 (24.2)	18 (23.7)	0.930	0.97 (0.52-1.82)
					AA	141 (75.8)	58 (76.3)		1
rs541671	1	p34.3	36905238	*GRIK3*	TT + TA	47 (26.1)	18 (25.3)	0.902	0.96 (0.51-1.80)
					AA	133 (73.9)	53 (74.7)		1
rs35317705	1	p34.3	36972969	*GRIK3*	CC + CT	22 (11.8)	11 (14.5)	0.558	1.26 (0.58-2.75)
					TT	164 (88.2)	65 (85.5)		1
rs11218005	11	q23.3	120782227	*GRIK4*	AA + AC	27 (14.5)	8 (10.5)	0.389	0.69 (0.30-1.60)
					CC	159 (85.5)	68 (89.5)		1
rs3901285	11	q23.3	120862726	*GRIK4*	TT + TC	113 (60.7)	45 (59.2)	0.817	0.94 (0.54-1.62)
					CC	73 (39.3)	31 (40.8)		1
rs4936566	11	q23.3	120944529	*GRIK4*	AA + AG	104 (55.9)	41 (54.0)	0.771	0.92 (0.54-1.58)
					GG	82 (44.1)	35 (46.0)		1
rs443239	19	q13.2	42001892	*GRIK5*	CC + CG	45 (24.2)	19 (25.0)	0.890	1.04 (0.56-1.94)
					GG	141 (75.8)	57 (75.0)		1
rs1493395	5	q33.2	153532297	*GRIA1*	AA + AG	88 (47.3)	37 (48.7)	0.840	1.06 (0.62-1.80)
					GG	98 (52.7)	39 (51.3)		1
rs12153489	5	q33.2	153568777	*GRIA1*	TT + CT	40 (21.5)	19 (25.0)	0.539	1.22 (0.65-2.28)
					CC	146 (78.5)	57 (75.0)		1
rs4424038	5	q33.2	153740704	*GRIA1*	TT + CT	23 (12.4)	10 (13.2)	0.861	1.07 (0.48-2.38)
					CC	163 (87.6)	66 (86.8)		1
rs17035909	4	q32.1	157247565	*GRIA2*	AA + AT	66 (35.7)	21 (27.6)	0.210	0.69 (0.38-1.24)
					TT	119 (64.3)	55 (72.4)		1
rs17035959	4	q32.1	157302204	*GRIA2*	CC + AC	72 (38.7)	25 (32.9)	0.376	0.78 (0.44-1.36)
					AA	114 (61.3)	51 (67.1)		1
rs7695870	4	q32.1	157342624	*GRIA2*	TT + CT	50 (26.9)	26 (34.2)	0.236	1.41 (0.80-2.51)
					CC	136 (73.1)	50 (65.8)		1
rs6855973	4	q32.1	157365463	*GRIA2*	TT + AT	60 (33.2)	18 (24.0)	0.148	0.64 (0.34-1.18)
					AA	121 (66.8)	57 (76.0)		1
rs10895875	11	q22.3	105785485	*GRIA4*	AA + AT	130 (69.9)	51 (67.1)	0.673	1.16 (0.58-2.30)
					TT	56 (30.1)	25 (32.9)		1
rs4754136	11	q22.3	105846312	*GRIA4*	TT + CT	26(14.0)	5 (6.6)	0.092	0.43 (0.16-1.17)
					CC	160 (86.0)	71 (93.4)		1
rs17104835	11	q22.3	105971356	*GRIA4*	CC + CT	66 (35.7)	38 (50)	***0.032***	***1.80*** **(** ***1.05***-***3.10*** **)**
					TT	119 (64.3)	38 (50)		***1***
rs7750018	6	q24.3	146206595	*GRM1*	CC + CT	43 (23.1)	19 (25.0)	0.745	1.11 (0.60-2.06)
					TT	143 (76.9)	57 (75.0)		1
rs362851	6	q24.3	146389448	*GRM1*	CC + CG	117 (62.9)	52 (68.4)	0.397	1.28 (0.72-2.25)
					GG	69 (37.1)	24 (31.6)		1
rs2300631	6	q24.3	146428918	*GRM1*	AA + AG	135 (72.7)	57 (75.0)	0.688	1.13 (0.62-2.09)
					GG	51 (27.4)	19 (25.0)		1
rs12023603	1	p21.2	51466999	*GRM2*	GG + AG	50 (26.9)	22 (28.9)	0.734	1.11 (0.61-2.00)
					AA	136 (73.1)	54 (71.1)		1
rs1983842	1	p21.2	51535259	*GRM2*	GG + AG	56 (30.1))	23 (30.2)	0.980	1.01 (0.56-1.80)
					AA	130 (69.9)	53 (69.8)		1
rs802441	7	q21.11	86657787	*GRM3*	TT + CT	51 (27.4)	23 (30.2)	0.643	1.15 (0.64-2.06)
					CC	135 (72.6)	53 (69.8)		1
rs802466	7	q21.11	86698122	*GRM3*	CC + CT	35 (18.8)	9 (11.8)	0.171	0.58 (0.26-1.27)
					TT	151 (81.2)	67 (88.2)		1
rs12704286	7	q21.11	86745625	*GRM3*	AA + AG	60 (33.3)	27 (38.6)	0.435	1.26 (0.71-2.23)
					GG	120 (66.7)	43 (61.4)		1
rs17697415	7	q21.11	86772500	*GRM3*	AA + AG	22 (11.8)	12 (15.8)	0.387	1.40 (0.65-2.99)
					GG	164 (88.2)	64 (84.2)		1
rs1873254	6	p21.31	34058712	*GRM4*	AA + AG	105 (57.4)	43 (57.3)	0.995	1.00 (0.58-1.72)
					GG	78 (42.6)	32 (42.7)		1
rs937039	6	p21.31	34075875	*GRM4*	GG + AG	44 (23.6)	17 (22.4)	0.823	0.93 (0.49-1.76)
					AA	142 (76.4)	59 (77.6)		1
rs1565361	6	p21.31	34089248	*GRM4*	CC + CT	84 (45.2)	33 (43.4)	0.797	0.93 (0.54-1.60)
					TT	102 (54.8)	43 (56.6)		1
rs4106126	11	q14.2	88647181	*GRM5*	TT + CT	43 (23.1)	18 (23.7)	0.922	1.03 (0.55-1.94)
					CC	143 (76.9)	58 (76.3)		1
rs1391878	11	q14.2	88713212	*GRM5*	CC + CT	39 (21.0)	19 (25.0)	0.476	1.26 (0.67-2.35)
					TT	147 (79.0)	57 (75.0)		1
rs12787863	11	q14.2	88810547	*GRM5*	AA + AG	77 (41.4)	33 (43.4)	0.763	1.09 (0.63-1.86)
					GG	109 (58.6)	43 (56.6)		1
rs7126679	11	q14.2	89020677	*GRM5*	AA + AG	109 (59.9)	48 (64.0)	0.539	1.19 (0.68-2.08)
					GG	73 (40.1)	27 (36.0)		1
rs2856354	5	q35.3	178978728	*GRM6*	GG + AG AA	81 (43.6)	37 (48.7)	0.448	1.23 (0.72-2.10)
						105 (56.4)	39 (51.3)		1
rs10464073	5	q35.3	178982284	*GRM6*	GG + AG	81 (43.6)	37 (48.7)	0.448	1.23 (0.72-2.10)
					AA	105 (56.4)	39 (51.3)		1
rs17078880	5	q35.3	178983436	*GRM6*	TT + CT	51 (27.7)	20 (26.7)	0.863	0.95 (0.52-1.74)
					CC	133 (72.3)	55 (73.3)		1
rs2645341	5	q35.3	178984314	*GRM6*	GG + AG	53 (28.5)	19 (25.0)	0.565	0.84 (0.45-1.54)
					AA	133 (71.5)	57 (75.0)		1
rs6764411	3	p26.1	7101864	*GRM7*	CC + AC	127 (68.3)	52 (68.4)	0.982	1.01 (0.57-1.79)
					AA	59 (31.7)	24 (31.6)		1
rs17697928	3	p26.1	7326084	*GRM7*	AA + AG	130 (69.9)	48 (63.2)	0.289	0.74 (0.42-1.29)
					GG	56 (30.1)	28 (36.8)		1
rs779741	3	p26.1	7541915	*GRM7*	CC + AC	124 (66.7)	48 (63.2)	0.587	0.86 (0.49-1.50)
					AA	62 (33.3)	28 (36.8)		1
rs1354405	3	p26.1	7690304	*GRM7*	GG + AG	103 (55.4)	38 (50.0)	0.428	0.81 (0.47-1.38)
					AA	83 (44.6)	38 (50.0)		1
rs712723	7	q31.33	126439090	*GRM8*	CC + CT	115 (61.8)	59 (77.6)	***0.014***	***2.14*** **(** ***1.16***-***3.6*** **)**
									1
rs17627206	7	q31.33	126793483	*GRM8*	TT AA + AG	71 (38.2) 20 (10.7)	17 (22.4) 8 (10.5)	0.957	0.98 (0.41-2.32)
					GG	166 (89.3)	68 (89.5)		1
rs11563505	7	q31.33	127059729	*GRM8*	TT + CT	46 (24.7)	19 (25.0)	0.964	1.01 (0.55-1.88)
					CC	140 (75.3)	57 (75.0)		1

Physical position of individual SNPs was based on the NCBI Assembly database: GRCH38 version.

GRIK, glutamate receptor, ionotropic, kainate; GRIA, glutamate receptor, ionotropic, AMPA; GRM, glutamate receptor, metabotropic, SNP, single nucleotide polymorphism; CI, confidence interval.

*p*-values were obtained by chi-square test (2 x 2 table).

Bold italic are significant at *p* value <0.05.

### Multivariate regression analyses shows that *GRIK1*genetic polymorphisms may be related to CAA formation in KD

According to the above results, statistically significant differences in factors associated with CAA formation in KD were noted for the clinical characteristics including fever duration (*p* <0.0001), first IVIG used time (*p* <0.0001; number of days after the first day of fever), and the *GRIK1* (rs466013, rs425507, rs38700, and rs402280) genetic variants (*p* =0.007, *p* =0.005, *p* =0.004, and *p* =0.022, respectively) (Tables 1 and3). To further confirm the genetic role of *GRIK1*, we used multivariate regression analyses to adjust those potential factors (i.e., fever duration and IVIG used time) that may affect the analysis. As shown in Table 3, significant associations between KD with CAA complications and the *GRIK1* (rs466013, rs425507, rs38700 and rs402280) genetic variants were observed (Full model: for rs466013: odds ratio = 2.12; 95% CI = 1.22-3.65; for rs425507: odds ratio = 2.16; 95% CI = 1.26-3.76; for rs388700: odds ratio = 2.16; 95% CI = 1.26-3.76; for rs402280: odds ratio = 1.89; 95% CI = 1.09-3.21). Taken together, these data suggest that the significant association observed between CAA complications and the presence of the *GRIK1* genotypes persists even after adjusting for the potential factors.

**Table 3:** **Association of** ***GRIK1*** **genetic polymorphisms with CAA complications in Taiwanese KD children by multivariate regression analysis**

***GRIK1***genetic polymorphisms	Odds ratio	95% CI	***p***value
**Full model (adjusted by fever duration and first IVIG used time)**			
rs466013	2.12	1.22-3.65	***0.011***
rs425507	2.16	1.26-3.76	***0.009***
rs388700	2.16	1.26-3.76	***0.009***
rs402280	1.89	1.09-3.21	***0.028***

*GRIK1*, glutamate receptor, ionotropic, kainate 1; IVIG, Intravenous immunoglobulin; CAA, Coronary artery aneurysm; CI, confidence interval.

Full model shows results from a logistic regression model including the indicated predictors, fever duration (days) and first IVIG used time (number of days after the first day of fever).

Bold italic are significant at *p* value <0.05.

## Discussion

Previous research from our lab suggests that the NMDA receptor (*GRIN3A*) from the glutamate receptor family may influence KD pathogenesis[26]. In this study, we screened the entire glutamate receptor family including the iGluRs and mGluRs (*GRIK*, *GRIA* and *GRM* gene families) and identified another member, namely *GRIK1*, that may be involved in the development of KD-associated CAA complications in Taiwanese children of Han Chinese ethnic background. The most striking finding of this study is that 4 *GRIK1* gene variants were found to be strongly associated with the presence of CAA in KD patients, even in the multivariable model.

Our genetic association study showed that none of the genes of the glutamate receptor gene family including *GRIK1*, *GRIK2*, *GRIK3*, *GRIK4*, *GRIK5*, *GRIA1*, *GRIA2*, *GRIA4*, *GRM1*, *GRM2*, *GRM3*, *GRM4*, *GRM5*, *GRM6*, *GRM7*, and *GRM8* genes contributed to KD susceptibility. However, genetic variation of the *GRIK1* locus may potential induce susceptibility to the development of KD with CAA complications. The significant association observed between KD with CAA complications and the *GRIK1* genetic variants (rs466013, rs425507, rs38700, and rs402280) was found to persist even after adjusting for fever duration and first IVIG used time. These results suggest that the *GRIK1* gene may be involved in CAA formation of KD. *GRIK1* polymorphisms have been investigation for their associations with different diseases including Juvenile absence epilepsy[28, 29], schizophrenia[30, 31], alcohol dependence[32], topiramate’s effects on heavy drinking[33, 34], topiramate-induced side effects[35], and hepatitis B virus (HBV)-related hepatocellular carcinoma[36]. However, these *GRIK1* polymorphism data of various studies are also not absolutely consistent and conclusive. These studies show that *GRIK1* gene may mainly contribute to neuropsychological diseases. Glutamate is known to signal and is released by nerves, macrophages, lymphocytes, and chondrocytes[37, 38]. These amino acids bind to iGluRs and mGluRs to regulate peripheral pain, release of cytokines and matrix metalloproteinases, and immune responses[39–41]. Our studies have firstly showed that glutamate receptors including NMDA[26] and KA receptors are involved in the CAA complications of KD regardless of the fever duration and first IVIG used time. KD is a multi-systemic disorder with a possible underlying pathology of immune-mediated vasculitis[1, 42]. The vascular endothelium forms a functional barrier between the vessel wall and the bloodstream. Recent studies have shown that regulation of certain glutamate receptors may induce the inflammation of endothelial cells, thereby mediating pathogenesis of vascular diseases[43, 44]. Although the current therapy for KD includes high doses of aspirin in conjunction with IVIG treatment[45], reports suggest that this regimen cannot efficiently prevent CAA development.

In this study, we showed that the glutamate receptor *GRIK1* is significantly associated with KD with CAA complications in Taiwanese children with Han Chinese ethnic background. Genetic polymorphisms of the *GRIK1* gene may play a role in KD pathogenesis and this molecule may serve as a therapeutic target for the KD treatment to prevent CAA development. Children with specific glutamate receptor genotypes related to KD should be careful assessed for CAA status at the time of diagnosis and monitored during the CAA development and related cardiovascular diseases. In addition to aspirin and IVIG therapy, the glutamate receptors may also serve as good targets for the design of novel KD therapeutics.

## Methods

### Patients

We performed a retrospective study. Individuals fulfilling the diagnostic criteria of KD (n = 262) were identified and enrolled into this study from the Department of Pediatrics at China Medical University Hospital in Taichung, Taiwan[46, 47]. This study population has been previously used for SNP analysis and KD studies[4, 10, 26, 48, 49]. In this study, there were 164 males and 94 females with an average age at diagnosis 1.75 ± 1.61 years. All the patients were diagnosed according to KD criteria[50]. All the patients underwent regular echocardiography examinations at the acute stage, 2 and 6 months after onset and once a year thereafter. CAA was identified when either the right or left coronary artery showed a dilated diameter of 3 mm in children younger than 5 years of age, or 4 mm in the older children[51]. According to the presence or absence of CAA, statistically significant differences between these 2 groups were found with respect to the fever duration and 1st IVIG used time (number of days after the first day of fever)[26, 48]. Only Han Chinese individuals, who account for 98% of the Taiwanese residents, were considered for recruitment. This study was approved by the Human Studies Committee of China Medical University Hospital (CMUH REC No. DMR101-IRB1-313 (CR-1)).

### Consent

The written informed consent was obtained from the patient’s guardian/parent/next of kin for the publication of this report and any accompanying images.

### SNP genotyping

Fifty-three single nucleotide polymorphisms (SNPs) of 16 genes within the glutamate receptor gene family including *GRIK1*, *GRIK2*, *GRIK3*, *GRIK4*, *GRIK5*, *GRIA1*, *GRIA2*, *GRIA4*, *GRM1*, *GRM2*, *GRM3*, *GRM4*, *GRM5*, *GRM6*, *GRM7*, and *GRM8* were selected from the NCBI SNP database and HAPMAP website (Tables 2 and3)[52]. Selection criteria for including SNPs in the analysis were a minimum allele frequency of >0.05 in the Han Chinese population and a Hardy-Weinberg equilibrium (*p* >0.05). A summary of information on the SNPs in the glutamate receptor genes (location, position, rs number, and genotype) is presented in Table 1. Briefly, genomic DNA was extracted from peripheral blood leukocytes according to the standard protocols (Genomic DNA kit; Qiagen). SNPs were genotyped using a custom-designed VeraCode GoldenGate Genotyping Assay System (Illumina); genotyping was performed as outlined inhttp://www.illumina.com/.

Primers and probes were designed and created using Custom VeraCode GoldenGate Genotyping Assay System software. Genotype calls were automatically generated using GenCall software version 3.1.3. We assessed the 8 VeraCode runs individually for intra-plate inconsistencies (e.g., variation in fluorescence intensities). Genotype cluster plots generated by individual VeraCode and SAM assays were visually inspected for call quality. Plots that appeared to be "unusually" clustered (i.e., unlike the predicted spread in terms of software-generated HWE or distance between clusters [θ]) were investigated further by selecting samples via direct Sanger sequencing for genotype confirmation. Samples were sequenced using Big Dye Terminator v3.1 (AB, Foster City, CA, USA) according to the manufacturer’s guidelines, and sequenced with an AB 3730 genetic analyzer.

### Analysis of haplotype blocks

Based on the HAPLOVIEW software, we used the Lewontin D’ measure to estimate the intermarker coefficient of LD of patients[53]. The confidence interval (CI) of LD was estimated using a resampling procedure and then used to construct the haplotype blocks.

### Statistical analyses

Data are expressed as means ± standard deviation for continuous variables. Genotypes were obtained by direct count, followed by allele frequency calculations (Table 1). χ^2^ tests were performed to determine the differences in categorical variables, and the odds ratio and 95% CI were calculated for the factors under consideration. Forward stepwise multivariate regression analyses were also performed to identify factors that contribute independently to CAA formation in KD. All statistical analyses were performed using SPSS (v12.0) for Windows.

## Electronic supplementary material

Additional file 1: Figure S1.: Search results of single nucleotide polymorphism (SNP) of rs466013 of the *GRIK1* gene used in this study (http://www.ncbi.nlm.nih.gov/projects/SNP/snp_ref.cgi?rs=466013). Above: Genomic location of rs466013 (pointed by red arrows; the NCBI Assembly database: GRCh37.p10 version). Down: Genomic location of rs466013 for 6 versions of the NCBI Assembly database (pointed by red arrows). **Figure S2.** Search results of single nucleotide polymorphisms (SNPs) of rs466013, rs425507, rs388700 and rs402280 of the *GRIK1* gene used in this study (http://genome.ucsc.edu/cgi-bin/hgTracks?db=hg18&position=chr21%3A30120300-30129700&hgsid=370279953_3haDCdtlwLEpPqkcmUFYdaAFYNhx). Above: Genomic location of the *GRIK1* gene. Down: Genomic location of rs466013, rs425507, rs388700 and rs402280 (pointed by red arrows; the NCBI Assembly database: NCBI36/hg18 version). **Figure S3.** *GRIK1* mRNA expression levels in peripheral blood mononuclear cells according to the *GRIK1* SNPs (rs388700 and rs402280) genotypes. The relative *GRIK1* expression was detected by quantitative real-time RT-PCR, and expression from individuals with TT + TA genotypes was compared to that from individuals with AA genotype. The *GRIK1* (NM_000830.3) primer sequences were 5′-gcggttagagatggatcaaca-3′ (located at nucleotide 2559–2579 of the transcript (NM_000830.3) and 5′-tcatgaaagcccacatcttct-3′ (located at nucleotide 2617–2637 of the transcript (NM_000830.3)). The relative expression levels were expressed as *GRIK1* mRNA/ *HPRT* mRNA ratio. **Figure S4.** Venn diagram of 4 GWAS studies. Gene SNPs from 4 GWAS studies were used for searching for common gene SNPs by using Venny website (http://bioinfogp.cnb.csic.es/tools/venny/). **Figure S5.** Venn diagram of 4 GWAS studies. Gene SNPs from 4 GWAS studies were used for searching for common gene SNPs by using Venny website (http://bioinfogp.cnb.csic.es/tools/venny/). **Figure S5**. Venn diagram of 4 GWAS studies. Gene SNPs from 4 GWAS studies were used for searching for common gene SNPs by using Venny website (http://bioinfogp.cnb.csic.es/tools/venny/). (PPT 1 MB)

Additional file 2: Table S1.: Characteristics of GWAS studies for KD susceptibility included in this meta-analysis. **Table S2.** Meta-analysis for previous reported GWAS studies for KD susceptibility. (DOC 54 KB)
