# Supplementary material for: Genetic variants of glutamate receptor gene family in Taiwanese Kawasaki disease children with coronary artery aneurysms
Source: Cell Biosci. 2014 Nov 19;4:67. doi: 10.1186/2045-3701-4-67 (PMC4258047; doi:10.1186/2045-3701-4-67)
Supplement: Supplementary file 1 — Additional file 1: Figure S1.: Search results of single nucleotide polymorphism (SNP) of rs466013 of the GRIK1 gene used in this study (http://www.ncbi.nlm.nih.gov/projects/SNP/snp_ref.cgi?rs=466013). Above: Genomic location of rs466013 (pointed by red arrows; the NCBI Assembly database: GRCh37.p10 version). Down: Genomic location of rs466013 for 6 versions of the NCBI Assembly database (pointed by red arrows). Figure S2. Search results of single nucleotide polymorphisms (SNPs) of rs466013, rs425507, rs388700 and rs402280 of the GRIK1 gene used in this study (http://genome.ucsc.edu/cgi-bin/hgTracks?db=hg18&position=chr21%3A30120300-30129700&hgsid=370279953_3haDCdtlwLEpPqkcmUFYdaAFYNhx). Above: Genomic location of the GRIK1 gene. Down: Genomic location of rs466013, rs425507, rs388700 and rs402280 (pointed by red arrows; the NCBI Assembly database: NCBI36/hg18 version). Figure S3. GRIK1 mRNA expression levels in peripheral blood mononuclear cells according to the GRIK1 SNPs (rs388700 and rs402280) genotypes. The relative GRIK1 expression was detected by quantitative real-time RT-PCR, and expression from individuals with TT + TA genotypes was compared to that from individuals with AA genotype. The GRIK1 (NM_000830.3) primer sequences were 5′-gcggttagagatggatcaaca-3′ (located at nucleotide 2559–2579 of the transcript (NM_000830.3) and 5′-tcatgaaagcccacatcttct-3′ (located at nucleotide 2617–2637 of the transcript (NM_000830.3)). The relative expression levels were expressed as GRIK1 mRNA/ HPRT mRNA ratio. Figure S4. Venn diagram of 4 GWAS studies. Gene SNPs from 4 GWAS studies were used for searching for common gene SNPs by using Venny website (http://bioinfogp.cnb.csic.es/tools/venny/). Figure S5. Venn diagram of 4 GWAS studies. Gene SNPs from 4 GWAS studies were used for searching for common gene SNPs by using Venny website (http://bioinfogp.cnb.csic.es/tools/venny/). Figure S5. Venn diagram of 4 GWAS studies. Gene SNPs from 4 GWAS studies were used for searching for c [file 13578_2014_193_MOESM1_ESM.ppt]

## Slide 1
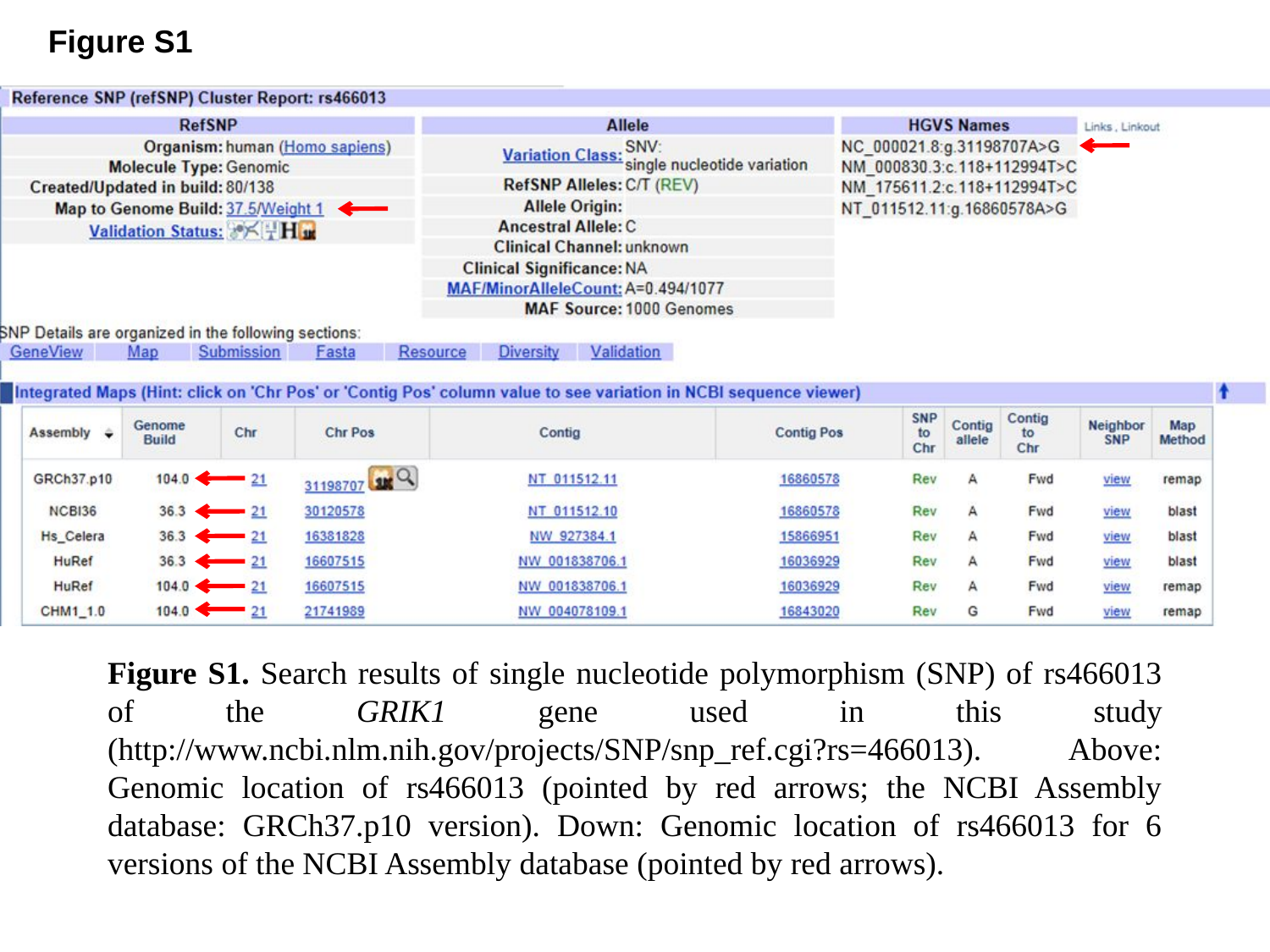

Figure S1
Figure S1. Search results of single nucleotide polymorphism (SNP) of rs466013 of the GRIK1 gene used in this study (http://www.ncbi.nlm.nih.gov/projects/SNP/snp_ref.cgi?rs=466013). Above: Genomic location of rs466013 (pointed by red arrows; the NCBI Assembly database: GRCh37.p10 version). Down: Genomic location of rs466013 for 6 versions of the NCBI Assembly database (pointed by red arrows).

## Slide 2
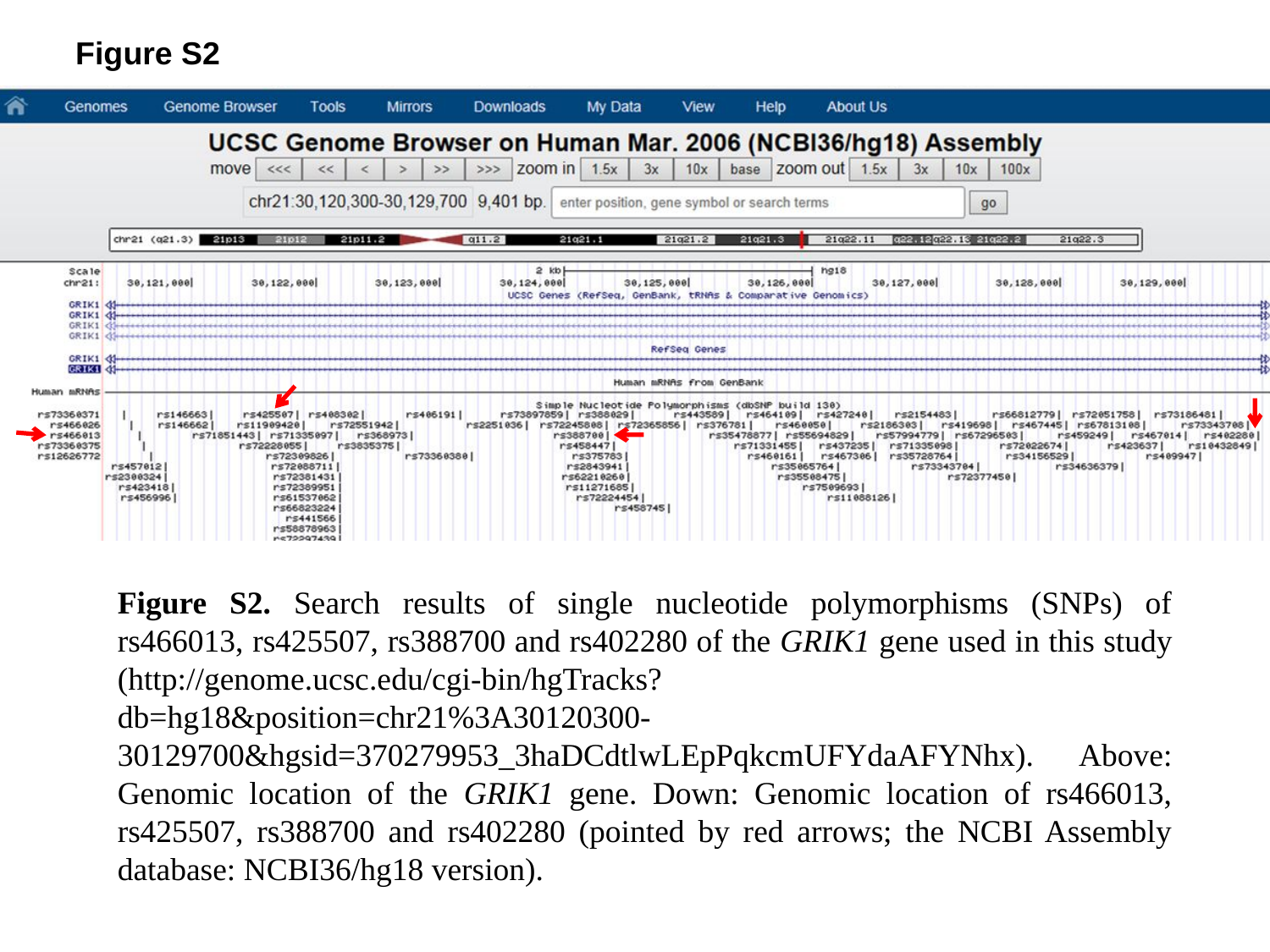

Figure S2
Figure S2. Search results of single nucleotide polymorphisms (SNPs) of rs466013, rs425507, rs388700 and rs402280 of the GRIK1 gene used in this study (http://genome.ucsc.edu/cgi-bin/hgTracks?db=hg18&position=chr21%3A30120300-30129700&hgsid=370279953_3haDCdtlwLEpPqkcmUFYdaAFYNhx). Above: Genomic location of the GRIK1 gene. Down: Genomic location of rs466013, rs425507, rs388700 and rs402280 (pointed by red arrows; the NCBI Assembly database: NCBI36/hg18 version).

## Slide 3
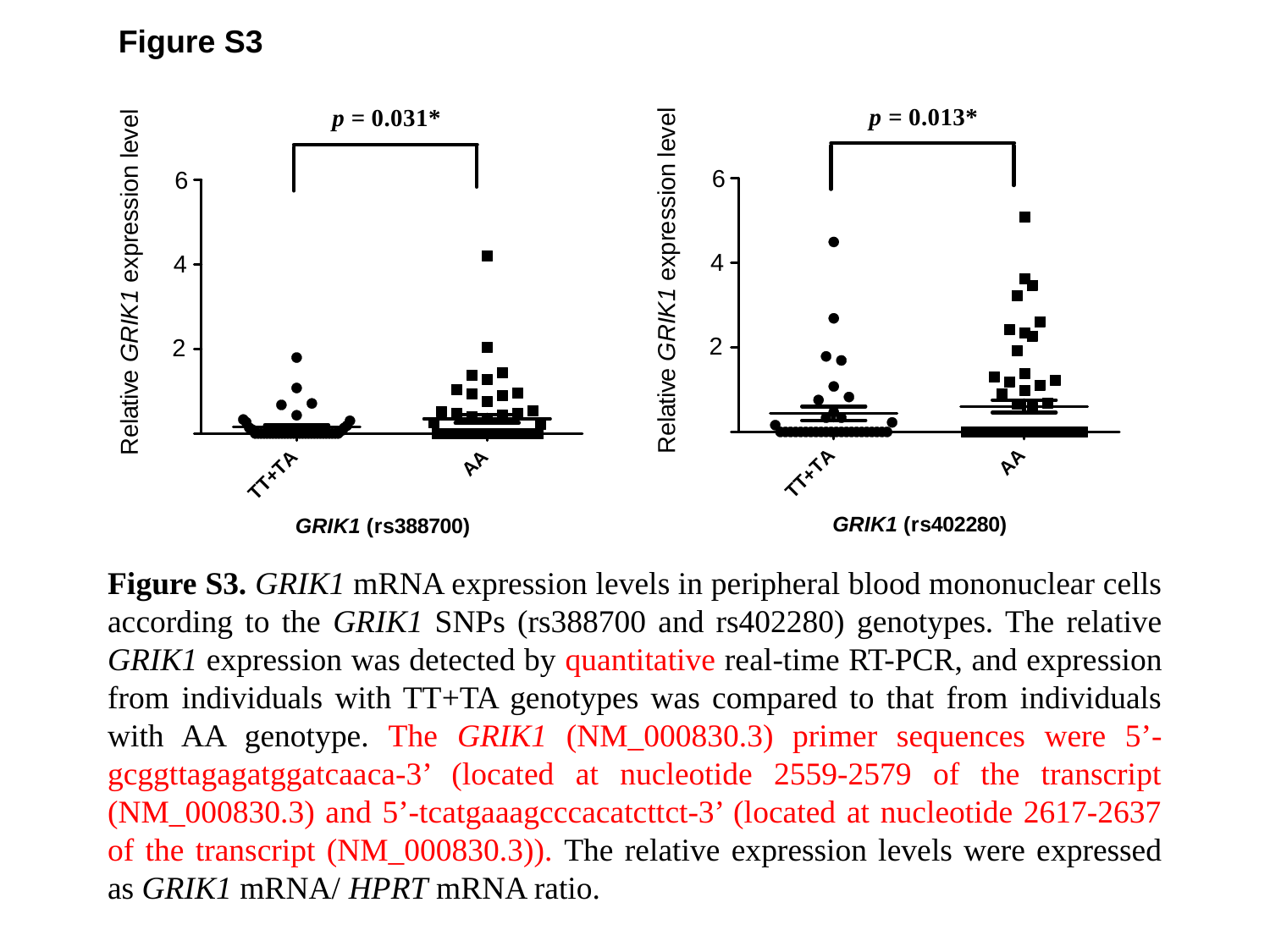

Figure S3
Figure S3. GRIK1 mRNA expression levels in peripheral blood mononuclear cells according to the GRIK1 SNPs (rs388700 and rs402280) genotypes. The relative GRIK1 expression was detected by quantitative real-time RT-PCR, and expression from individuals with TT+TA genotypes was compared to that from individuals with AA genotype. The GRIK1 (NM_000830.3) primer sequences were 5’-gcggttagagatggatcaaca-3’ (located at nucleotide 2559-2579 of the transcript (NM_000830.3) and 5’-tcatgaaagcccacatcttct-3’ (located at nucleotide 2617-2637 of the transcript (NM_000830.3)). The relative expression levels were expressed as GRIK1 mRNA/ HPRT mRNA ratio.

## Slide 4
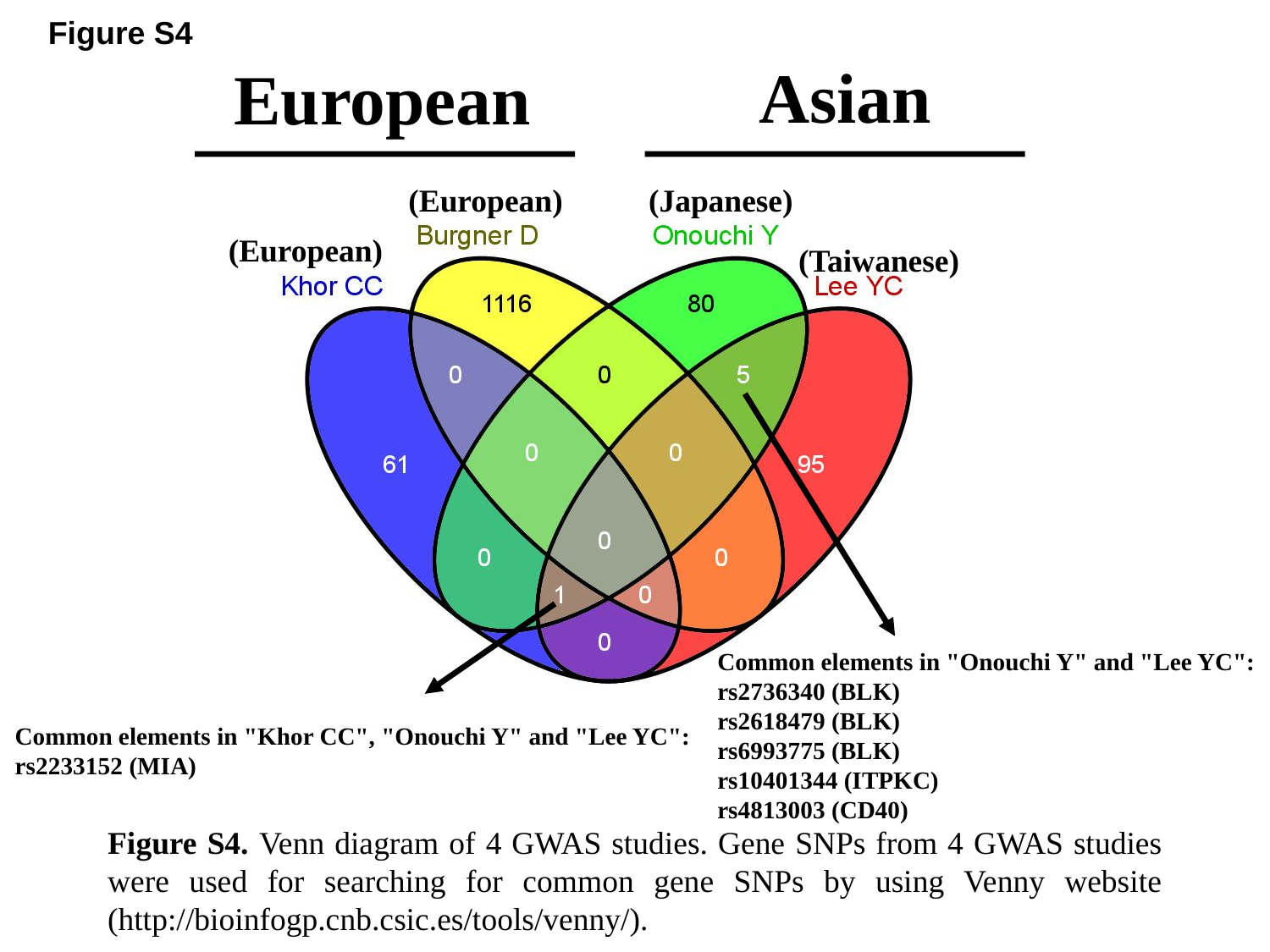

Figure S4
Asian
European
(European)
(Japanese)
(European)
(Taiwanese)
Common elements in "Onouchi Y" and "Lee YC":
rs2736340 (BLK)
rs2618479 (BLK)
rs6993775 (BLK)
rs10401344 (ITPKC)
rs4813003 (CD40)
Common elements in "Khor CC", "Onouchi Y" and "Lee YC":
rs2233152 (MIA)
Figure S4. Venn diagram of 4 GWAS studies. Gene SNPs from 4 GWAS studies were used for searching for common gene SNPs by using Venny website (http://bioinfogp.cnb.csic.es/tools/venny/).

## Slide 5
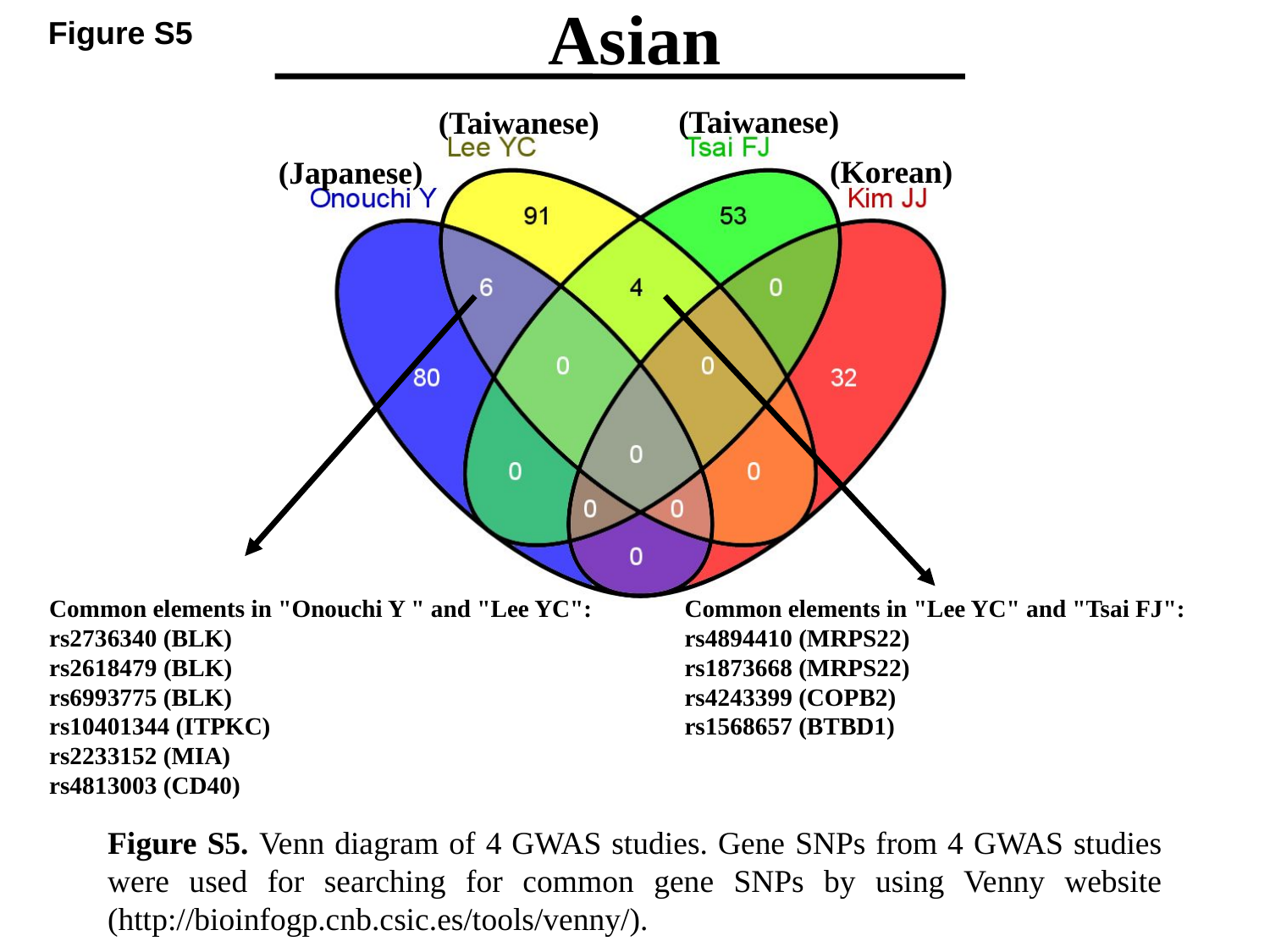

Asian
Figure S5
(Taiwanese)
(Taiwanese)
(Korean)
(Japanese)
Common elements in "Onouchi Y " and "Lee YC":
rs2736340 (BLK)
rs2618479 (BLK)
rs6993775 (BLK)
rs10401344 (ITPKC)
rs2233152 (MIA)
rs4813003 (CD40)
Common elements in "Lee YC" and "Tsai FJ":
rs4894410 (MRPS22)
rs1873668 (MRPS22)
rs4243399 (COPB2)
rs1568657 (BTBD1)
Figure S5. Venn diagram of 4 GWAS studies. Gene SNPs from 4 GWAS studies were used for searching for common gene SNPs by using Venny website (http://bioinfogp.cnb.csic.es/tools/venny/).
