# Supplementary material for: Genetic variants of glutamate receptor gene family in Taiwanese Kawasaki disease children with coronary artery aneurysms
Source: Cell Biosci. 2014 Nov 19;4:67. doi: 10.1186/2045-3701-4-67 (PMC4258047; doi:10.1186/2045-3701-4-67)
Supplement: Supplementary file 2 — Additional file 2: Table S1.: Characteristics of GWAS studies for KD susceptibility included in this meta-analysis. Table S2. Meta-analysis for previous reported GWAS studies for KD susceptibility. (DOC 54 KB) [file 13578_2014_193_MOESM2_ESM.doc]

| **Table S1 Characteristics of GWAS studies for KD susceptibility included in this meta-analysis** | | | | | |  |  |  |  |
| --- | --- | --- | --- | --- | --- | --- | --- | --- | --- |
| **Reference** |  | **No. of SNPs** | **Year** | **Population** | **GWAS** | |  | **Follow-up study** | |
|  |  |  |  |  | **Case (Number)** | **Control (Number)** |  | **Case (Number)** | **Control (Number)** |
|  |  |  |  |  |  |  |  |  |  |
| Onouchi Y et al., 2012 |  | 473803 | 2012 | Japanese | 428 | 3379 |  | 754 | 947 |
| Lee YC et al., 2012 |  | 716935 | 2012 | Taiwanese | 622 | 1107 |  | 261 | 550 |
| Khor CC et al., 2011 |  | 494236 | 2011 | European | 405 | 6252 |  | - | - |
| Khor CC et al., 2011 |  | 494236 | 2011 | Taiwanese | - | - |  | 438 | 446 |
| Khor CC et al., 2011 |  | 494236 | 2011 | Korean | - | - |  | 460 | 498 |
| Khor CC et al., 2011 |  | 494236 | 2011 | Hong Kong and Shanghai | - | - |  | 130 | 568 |
| Tsai FJ et al., 2011 |  | 723638 | 2011 | Taiwanese | 250 | 446 |  | 208 | 366 |
| Kim JJ et al., 2011 |  | 641760 | 2011 | Korean | 186 | 600 |  | 514 | 1042 |
| Burgner D et al., 2009 |  | 223922 | 2009 | European | 119 | 135 |  | - | - |

| Table S2 Meta-analysis for previous reported GWAS studies for KD susceptibility | | | | | | |  |  |  |  |
| --- | --- | --- | --- | --- | --- | --- | --- | --- | --- | --- |
| Gene | Variant | Risk allele | Total case subjects with risk allele(%) | Total control subjects with risk allele(%) | Pooled OR | (95% CI) | Number of studies | Heterogeneity | | |
|  |  |  |  |  |  |  |  | I^2^ | x^2^ | *p* value |
| *ITPKC* | rs10401344 | T | 1804 (18.7) | 5433 (13.7) | 1.47 | (1.32- 1.65) | 2 | 0 | 2.35 | 0.5032 |
| *MIA* | rs2233152 | A | 2209 (18.5) | 11685 (13.1) | 1.54 | (1.38- 1.73) | 3 | 0 | 2.32 | 0.5088 |
| *BLK* | rs2618479 | C | 1804 (79.4) | 5433 (70.4) | 1.6 | (1.46- 1.77) | 2 | 18.66 | 3.69 | 0.2972 |
| *BLK* | rs2736340 | T | 1804 (79.0) | 5433 (69.8) | 1.62 | (1.48- 1.77) | 2 | 0 | 1.59 | 0.8115 |
| *CD40* | rs4813003 | G | 1804 (71.0) | 5433 (64.7) | 1.35 | (1.24- 1.48) | 2 | 0 | 0.44 | 0.9326 |
| *BLK* | rs6993775 | T | 1804 (78.2) | 5433 (70.3) | 1.52 | (1.38- 1.67) | 2 | 7.24 | 3.23 | 0.3569 |
